# Target trial on the outcomes of laparoscopic compared to robotic-assisted proctectomy in stage II–III rectal cancer

**DOI:** 10.1007/s13304-025-02446-0

**Published:** 2025-10-16

**Authors:** Sameh Hany Emile, Nir Horesh, Marcus Oosenbrug, Ebram Salama, Anjelli Wignakumar, Victor Strassmann, Steven D. Wexner

**Affiliations:** 1 Colorectal Surgery Department, Cleveland Clinic Florida, Ellen Leifer Shulman and Steven Shulman Digestive Disease Center, 2950 Cleveland Clinic Blvd., Weston, FL 33179 USA; 2 Colorectal Surgery Unit, General Surgery Department, Mansoura University Hospitals, Mansoura, Egypt; 3 Department of Surgery and Transplantations, Ramat Gan and Faculty of Medicine, Sheba Medical Center, Tel Aviv University, Tel Aviv, Israel

**Keywords:** Target trial, Outcomes, Laparoscopic, Robotic-assisted, Proctectomy, Rectal cancer

## Abstract

Although outcomes of laparoscopic and robotic-assisted proctectomy have been compared, the superiority of one approach over another was not proven. We used the target trial methodology to emulate a randomized clinical trial comparing laparoscopic and robotic-assisted proctectomy for rectal cancer. Data from the NCDB (2015–2021) on patients with stage II–III rectal adenocarcinoma were collected. Patients were divided into two groups: laparoscopic proctectomy (LP) and robotic-assisted proctectomy (RP). The groups were matched for baseline patient and treatment confounders to obtain balanced groups, emulating the design of a randomized trial. The primary outcome was the pathologic outcomes of each surgical approach, including the status of circumferential resection margins (CRM), surgical margins, and number of examined lymph nodes. Secondary outcomes included conversion to open surgery, hospital stay, and 30- and 90-day mortality. After matching, 5631 patients were included in each group. The RP and LP groups had similar rates of positive CRM (10.5% vs. 9.4%, *p* = 0.072), positive surgical margins (6% vs. 6.3%, *p* = 0.528), examined lymph nodes number (median: 15 vs. 15, *p* = 0.105), 30-day mortality (0.7% vs. 0.9%, *p* = 0.405), 90-day mortality (1.5% vs. 1.8%, *p* = 0.333), and unplanned 30-day readmission (6.6% vs. 6.2%, *p* = 0.477). RP was associated with shorter hospital stay (median: 4 vs. 5 days, *p* < 0.001) and lower rate of conversion to open surgery (5.8% vs. 13.6%, OR: 0.39, 95%CI 0.34–0.45, *p* < 0.001). This target trial found RP and LP for rectal cancer associated with similar clinical and pathologic outcomes. RP was associated with fewer conversions and shorter hospital stays than LP.

**Supplementary Information:**

The online version contains supplementary material available at 10.1007/s13304-025-02446-0.

## Introduction

Surgery for locally advanced rectal cancer is technically demanding and requires both extensive and continued learning and ideally high-volume practice [1]. Rectal cancer resection can be particularly challenging in males with increased body mass index and after neoadjuvant radiation therapy [2]. Recent technology has facilitated complex and technically challenging minimally invasive pelvic procedures, including three-dimensional cameras, articulated instruments, near-infrared fluorescence angiography, and robotic-assisted surgery [3, 4].

The robotic-assisted platform is perhaps one of the most important innovations used in rectal cancer surgery. Claimed advantages of the robotic platform include the highly magnified three-dimensional vision of the pelvis, improved ergonomics, precision in the surgical instrument control, and a greater range of motion and dexterity [5]. However, these technical aspects may not translate into tangible patient clinical benefits. After both approaches, evidence from randomized clinical trials comparing robotic-assisted to laparoscopic proctectomy showed similar pathologic outcomes [6], bowel function recovery, and quality of life [7]. Even the presumed benefit of the robotic platform in reducing conversion rates shown in observational studies [8], was not substantiated in randomized trials [9, 10]. Although the REAL trial [11] concluded significant benefits of robotic-assisted surgery for rectal cancer, including lower rates of positive circumferential resection margin, more complete mesorectal excisions, and fewer conversions to open surgery than laparoscopic surgery, the difference in these outcomes was around 2–3% which may not be clinically relevant.

Apart from the REAL multicenter trial [11], other randomized trials [6, 7, 10] comparing robotic and laparoscopic rectal cancer surgery did not have sufficient sample size to ensure adequate study power. Specifically, the COLRAR trial [6] was prematurely terminated because of poor data accrual. Given the logistic challenges in patient recruitment, securing funds, and follow-ups, alternative solutions have been proposed. Recently, the target trial, a hypothetical randomized trial, was described. This hypothetical trial uses big observational data to mimic the design of a randomized trial that is otherwise not practical to conduct due to high costs, poor feasibility, and being time-consuming [12]. Therefore, the present study used the target trial methodology, aiming to emulate a randomized clinical trial comparing laparoscopic and robotic-assisted proctectomy for rectal cancer.

## Patients and methods

### Study design

The protocol of a target trial on rectal cancer surgery has been specified before conducting this study (Table 1). Prospectively collected data from the National Cancer Database (NCDB) were used to emulate a target trial [12] that compared robotic-assisted (treatment arm) and laparoscopic (control arm) proctectomy for locally advanced rectal cancer. The study was not considered human subject research as it was a retrospective review of de-identified data from a national database; therefore, ethics approval was not required. Given the retrospective nature of the study, we did not involve patients or the public in the design, conduct, or reporting of the study. The study was reported in adherence to the STROBE guidelines.

**Table 1:** Target trial protocol

Component	Target trial	Emulated trial using real-world data
Objective	To compare the pathologic outcomes of laparoscopic and robotic-assisted proctectomy of rectal cancer	Same
Population	Adult patients aged > 18 years of either sex with clinical stage II–III rectal adenocarcinoma confirmed by histologic examination of the specimen after proctectomy	Same
Treatment strategies	Laparoscopic proctectomy Robotic-assisted proctectomy	Same
Treatment assignment	Random allocation to each treatment strategy	The assignment was not random and was on a case-by-case basis at the discretion of the surgeons and patient preference. Possible criteria that guided the selection of the surgical approach include patient demographics, tumor stage, location and size, and logistic considerations related to insurance state,facility type, and availability of laparoscopic and robotic platforms. Randomization was emulated by adjusting for baseline variables using the propensity-score matched method
Follow-up	Follow-up started from the date of proctectomy (zero) until the event or loss to follow-up, whichever occurred first Patients were classified into either group based on the surgical approach used at the start of the procedure, regardless of whether it was successfully completed with a minimally invasive approach or was converted to an open surgery	Same
Outcome	Pathologic outcome of each surgical approach, which included the status of circumferential resection margins, surgical margins, and the number of examined lymph nodes	Same
Causal contrast	The intention-to-treat effect, (effect of being assigned to laparoscopic or robotic-assisted proctectomy at baseline, regardless of whether the procedure was completed as planned)	Same. Patients converted to open surgery in each group were not excluded from the analysis
Statistical analysis	Intention-to-treat analysis,	Same

### Data sources

Data were collected from the NCDB between 2015 and 2021. The NCDB is a comprehensive national database that includes prospectively maintained data from more than 1500 hospitals accredited by the Commission on Cancer (CoC). It is a joint project of the Commission on Cancer (CoC) of the American College of Surgeons and the American Cancer Society. The NCDB and its participating hospitals are not responsible for the statistical validity of the analysis or the study's conclusions.

### Study population

The study included adult patients aged > 18 years of either sex with clinical stage II–III rectal adenocarcinoma confirmed by histologic examination of the specimen after proctectomy. Adenocarcinomas, mucinous adenocarcinomas, and signet-ring cell carcinomas were identified from the database using the ICDO-3 codes: 8140/3, 8480-8481/3, 8490/3. Exclusions were made for patients with stage I, stage IV, or unknown clinical stage, patients who underwent local tumor excision, total proctocolectomy, or non-specified surgery, and patients who underwent open proctectomy or with an unknown surgical approach.

### Treatment allocation

Since the data used in the study are retrospective, the assignment of patients to either laparoscopic or robotic-assisted proctectomy was not random and likely was at the discretion of the surgeons and patient preference, guided by patient demographics, tumor stage, location, and size, in addition to logistic considerations related to surgeon experience, insurance, facility type, and availability of laparoscopy and robotic platforms.

### Follow-up

The start point of follow-up (baseline) was the day of proctectomy. Patients were classified as laparoscopic or robotic-assisted proctectomy based on the surgical approach used at the start of the procedure, regardless of whether it was successfully completed with a minimally invasive approach or was converted to open surgery. Follow-up for clinical outcomes was conducted from baseline until the event or loss to follow-up, whichever occurred first.

### Study outcomes

The primary outcome was the pathologic outcomes of each surgical approach, including the status of circumferential resection margins (CRM), surgical margins, and the number of examined lymph nodes. CRM was considered positive when cancer cells were detected at or within 1 mm of the radial margin of the surgical resection. Surgical margins were considered positive if microscopic or macroscopic tumor residues were detected at the proximal, distal, or radial resection margins. Secondary outcomes included conversion to open surgery, hospital stay, 30-day and 90-day mortality as a surrogate for major complications, 30-day readmission, and 5-year overall survival calculated from surgery to death from any cause or loss to follow-up. 30-day readmission was classified as planned and unplanned. Unplanned readmission indicates a readmission to address a surgical or medical complication that occurred within 30 days after discharge. Planned readmission indicates readmission for a scheduled procedure, such as closure of a loop ileostomy, after the index surgery.

### Statistical analysis

Statistical analyses were performed using EZR (version 1.55) and R software (version 4.1.2). Depending on the distribution, continuous variables were expressed as mean ± standard deviation or median and interquartile range (IQR). Student’s t-test or Mann–Whitney U test was used to process continuous data, as appropriate. Categorical data, expressed as counts and percentages, were analyzed using Fisher's exact or Chi-square tests. Complete case analysis was used to handle missing data. To account for the lack of randomization, a 1:1 nearest neighborhood propensity score matching with a caliper of 0.2 was used to balance the two groups regarding differences in baseline variables. Variables of the propensity score model were determined before any outcome analysis. Based on the available baseline data, a propensity score model included clinically relevant covariates that exhibited significant imbalance at baseline. The standardized mean difference (SMD) was used to define clinically significant imbalance at a threshold of 0.1. An SMD < 0.1 indicates well-balanced groups. The propensity score reflected the probability of each patient being assigned to the laparoscopic or robotic-assisted groups, based on observed covariates, matching patients with similar propensity scores in each group. 5-year overall survival rates with their respective confidence intervals (CIs) were calculated with Kaplan–Meier statistics and Cox proportional hazards models were used to determine the predictors of OS. Sensitivity analyses based on disease stage and type of surgery were performed.

## Results

### Characteristics of patients

**Fig. 1:**
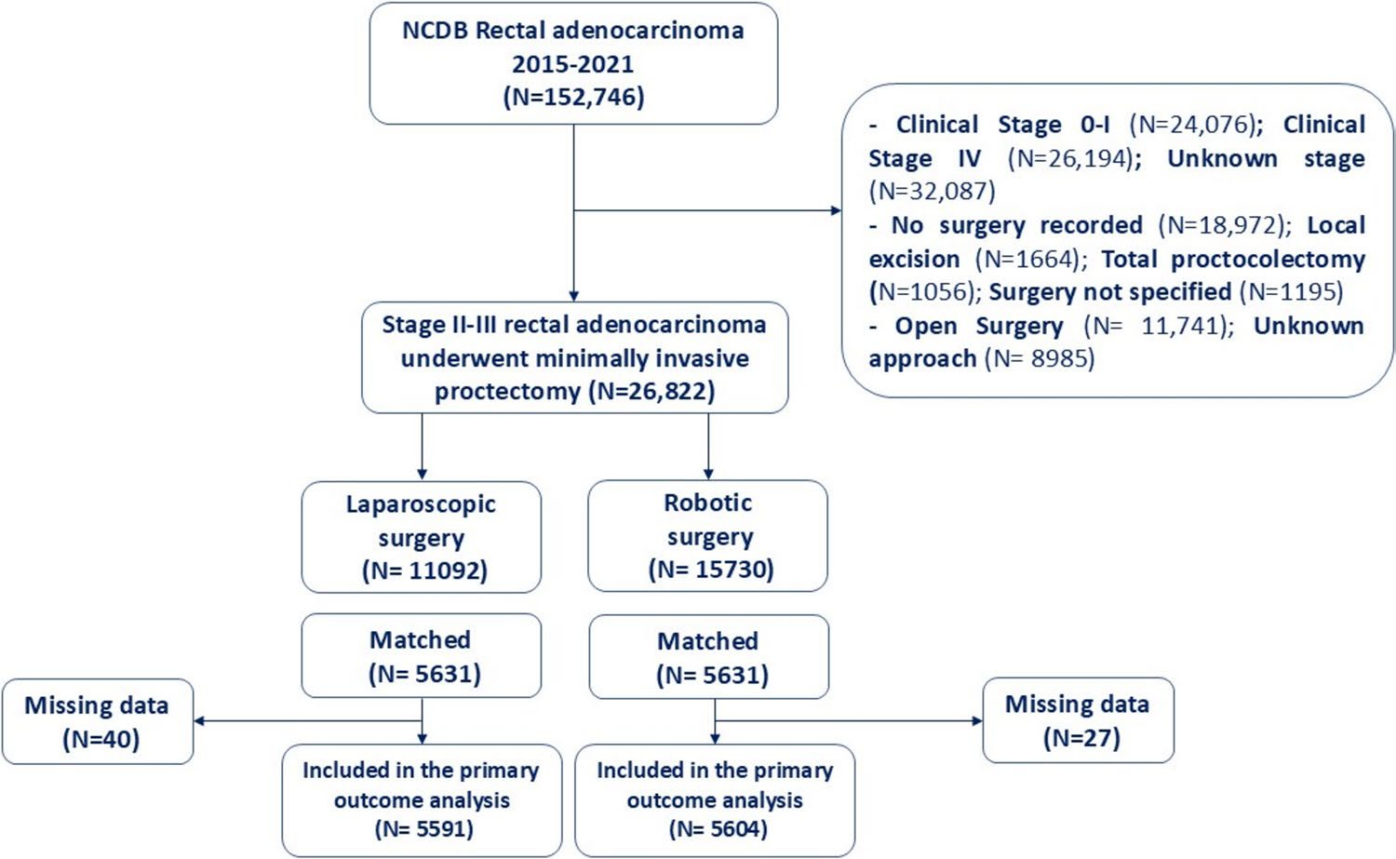
Flow chart for patient inclusion

A total of 152,746 patients with rectal adenocarcinomas were screened; 26,822 patients who met the inclusion criteria for the study were enrolled (Fig. 1). The mean age of patients was 60 years. Most patients were male (62%), white (84.9%), of Charlson score 0 (77.5%), had private insurance (51.9%), and were treated at academic or research programs (38.9%). 65.6% of patients had stage III disease and 34.4% had stage II. Neoadjuvant radiation therapy was given to 84.6% of patients and 70.4% underwent low anterior resection whereas 20.4% underwent abdominoperineal resection (Supplementary Table).

### Matching

Robotic-assisted proctectomy was performed in 15,730 (58.6%) and laparoscopic proctectomy in 11,092 (43.4%) patients. Patients who had robotic-assisted proctectomy more often were male (62.9% vs. 60.8%), had stage III disease (66.2% vs. 64.7%), private insurance (53.3% vs. 50%), elevated pretreatment CEA levels (67.9% vs. 60.6%), were treated at integrated network cancer programs (24.5% vs. 19%), received neoadjuvant radiation (86% vs. 82.7%), and had a longer time between diagnosis and surgery (161 vs. 141 days) (Table 2).

**Table 2:** Characteristics of patients before and after matching

		Before matching			After matching		
Factor	Group	Laparoscopic	Robotic	SMD	Laparoscopic	Robotic	SMD
Number		11,092	15,730		5631	5631	
Mean age in years (SD)		60.37 (12.57)	59.78 (12.30)	0.047	61.49 (11.13)	61.10 (10.89)	0.036
Sex (%)	Male	6739 (60.8)	9889 (62.9)	0.043	3381 (60.0)	3566 (63.3)	0.068
	Female	4353 (39.2)	5841 (37.1)		2250 (40.0)	2065 (36.7)	
Race (%)	White	9324 (84.7)	13,252 (84.9)	0.032	4688 (83.8)	4734 (84.6)	0.043
	Black	900 (8.2)	1176 (7.5)		489 (8.7)	440 (7.9)	
	Asian	532 (4.8)	829 (5.3)		279 (5.0)	302 (5.4)	
	American Indian	66 (0.6)	84 (0.5)		30 (0.5)	24 (0.4)	
	Other	191 (1.7)	269 (1.7)		105 (1.9)	93 (1.7)	
Ethnicity (%)	Hispanic	903 (8.3)	1255 (8.1)	0.006	413 (7.4)	358 (6.4)	0.039
	Non-hispanic	10,019 (91.7)	14,225 (91.9)		5135 (92.6)	5193 (93.6)	
Charlson Deyo score (%)	0	8596 (77.5)	12,181 (77.4)	0.016	4254 (75.5)	4331 (76.9)	0.034
	1	1698 (15.3)	2439 (15.5)		943 (16.7)	879 (15.6)	
	2	455 (4.1)	663 (4.2)		258 (4.6)	255 (4.5)	
	3	343 (3.1)	447 (2.8)		176 (3.1)	166 (2.9)	
Residence (%)	Metropolitan	8823 (82.1)	12,430 (81.9)	0.005	4467 (81.8)	4386 (81.0)	0.023
	Urban	1718 (16.0)	2451 (16.1)		891 (16.3)	931 (17.2)	
	Rural	212 (2.0)	298 (2.0)		101 (1.9)	99 (1.8)	
Median household income %)	> $63,000	3193 (34.3)	4684 (35.1)	0.022	1578 (33.4)	1669 (35.0)	0.039
	$48,000–$62,999	2536 (27.2)	3654 (27.4)		1299 (27.5)	1284 (26.9)	
	$38,000–$47,999	2116 (22.7)	2998 (22.4)		1083 (22.9)	1093 (22.9)	
	< $38,000	1471 (15.8)	2021 (15.1)		763 (16.2)	723 (15.2)	
Insurance type (%)	Medicaid	1053 (9.6)	1385 (8.9)	0.072	546 (9.8)	462 (8.3)	0.095
	Medicare	3962 (36.0)	5317 (34.1)		2072 (37.1)	1984 (35.6)	
	Other Government	147 (1.3)	203 (1.3)		71 (1.3)	62 (1.1)	
	Private	5504 (50.0)	8302 (53.3)		2721 (48.7)	2935 (52.7)	
	Not insured	336 (3.1)	374 (2.4)		179 (3.2)	130 (2.3)	
Facility type (%)	Academic/research program	4323 (40.9)	5597 (37.5)	**0.137**	2304 (40.9)	2230 (39.6)	0.077
	Community cancer program	368 (3.5)	410 (2.7)		146 (2.6)	151 (2.7)	
	Comprehensive community cancer program	3860 (36.5)	5263 (35.3)		2082 (37.0)	1978 (35.1)	
	Integrated network Cancer program	2012 (19.0)	3651 (24.5)		1099 (19.5)	1272 (22.6)	
Clinical TNM stage (%)	II	3917 (35.3)	5319 (33.8)	0.032	1931 (34.3)	1929 (34.3)	0.001
	III	7175 (64.7)	10,411 (66.2)		3700 (65.7)	3702 (65.7)	
Tumor histology (%)	Adenocarcinoma	10,698 (96.4)	15,188 (96.6)	0.011	5410 (96.1)	5395 (95.8)	0.015
	Mucinous adenocarcinoma	342 (3.1)	461 (2.9)		190 (3.4)	200 (3.6)	
	Signet ring cell carcinoma	52 (0.5)	81 (0.5)		31 (0.6)	36 (0.6)	
Tumor Grade (%)	Low	8044 (89.8)	11,162 (90.3)	0.017	4249 (89.4)	4315 (90.2)	0.027
	High	917 (10.2)	1203 (9.7)		504 (10.6)	468 (9.8)	
Median tumor size in mm [IQR]		45[30, 60]	46 [30, 60]	0.022	46 [30, 60]	47 [30, 60]	0.025
Pretreatment CEA levels (%)	Normal	2401 (38.6)	2433 (31.0)	**0.16**	2063 (36.6)	2274 (40.4)	0.077
	Borderline	49 (0.8)	86 (1.1)		46 (0.8)	45 (0.8)	
	Elevated	3775 (60.6)	5324 (67.9)		3522 (62.5)	3312 (58.8)	
Neoadjuvant radiation (%)	No	1889 (17.3)	2177 (14.0)	0.093	827 (15.0)	753 (13.5)	0.042
	Yes	9000 (82.7)	13,416 (86.0)		4684 (85.0)	4807 (86.5)	
Systemic therapy (%)	No	894 (8.1)	960 (6.1)	0.161	384 (6.8)	340 (6.0)	0.057
	Neoadjuvant	5861 (52.9)	9413 (59.9)		2944 (52.4)	2982 (53.0)	
	Adjuvant	4312 (39.0)	5330 (34.0)		2293 (40.8)	2302 (41.0)	
Median time before surgery in days [IQR]		147[124, 195]	161[132, 243]	**0.251**	147[126, 184	147[126, 183]	0.002
Median duration of radiation in days [IQR]		39 [35, 42]	39 [36, 42]	0.055	39[36, 42]	39[36, 42]	0.032
Type of surgery (%)	Low anterior resection	8014 (72.3)	10,856 (69.0)	0.091	3986 (70.8)	3783 (67.2)	0.086
	Abdominoperineal resection	2026 (18.3)	3445 (21.9)		1100 (19.5)	1290 (22.9)	
	Pelvic exenteration	226 (2.0)	318 (2.0)		119 (2.1)	120 (2.1)	
	Pull through with coloanal anastomosis	826 (7.4)	1111 (7.1)		426 (7.6)	438 (7.8)	

CEA, carcinoembryonic antigen; SMD, standard mean difference; IQR, interquartile range. Bold text in SMD columns indicates statistical significance

**Fig. 2:**
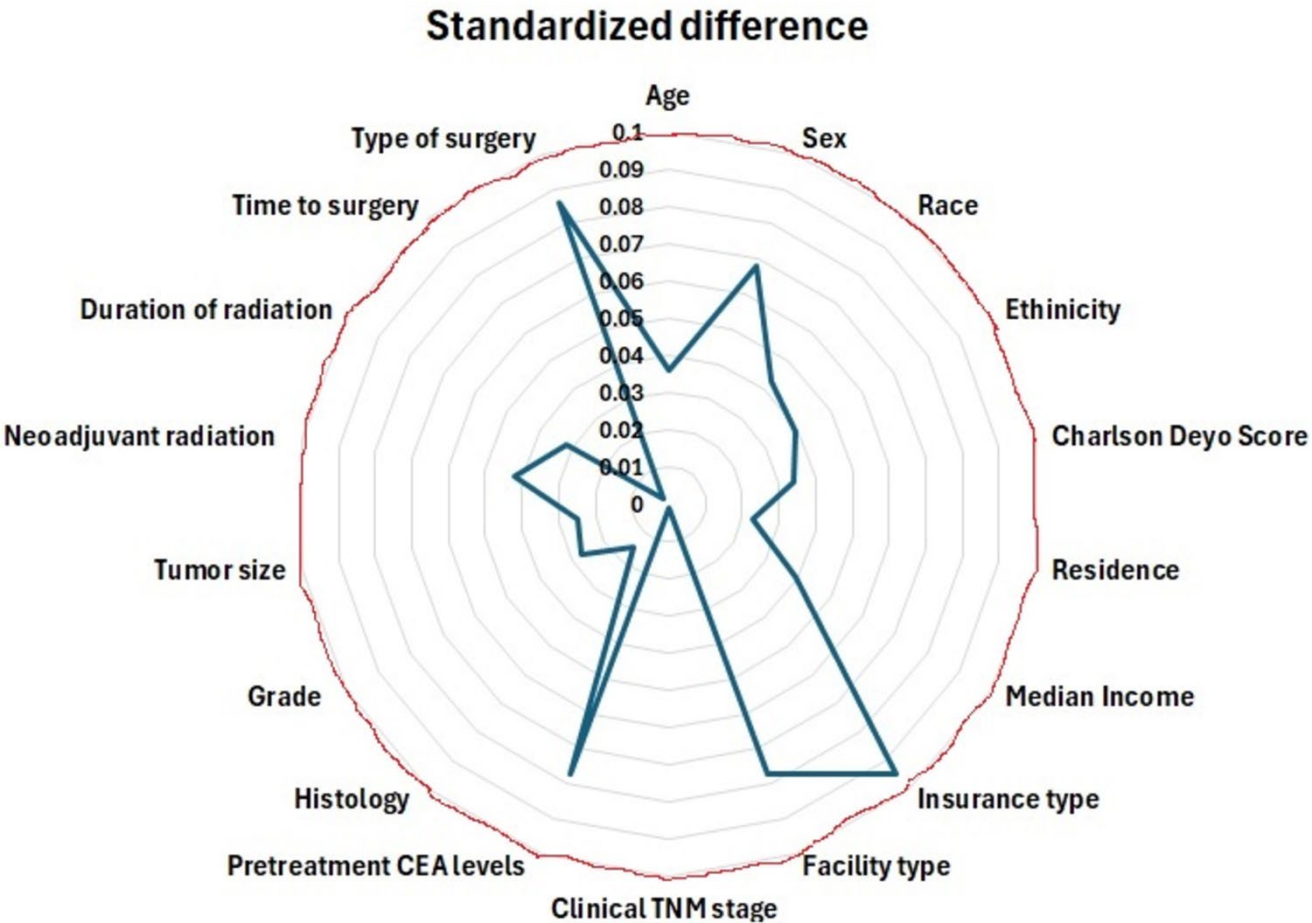
Plot showing an adequate balance of robotic-assisted and laparoscopic proctectomy for baseline variables with standardized differences < 0.1

After propensity score matching, 5631 patients were included in each group. The propensity scores ranged from 0.25 to 0.9 in the laparoscopic group and from 0.25 to 0.99 in the robotic group, with 97.5% in the region of common support (propensity scores, 0.2–0.99)). After propensity-score matching, all covariates had standardized mean differences < 10%, indicating an adequate balance of the two groups (Fig. 2).

### Primary outcome

The robotic and laparoscopic groups had similar rates of positive CRM (10.5% vs. 9.4%, OR: 1.13, 95%CI 0.99–1.26, *p* = 0.072), positive surgical margins (10.6% vs. 11.3%, OR: 0.94, 95%CI 0.83, 1.05, *p* = 0.307), and examined lymph nodes number (median: 15 vs. 15, *p* = 0.105). The median CRM was similar between the two approaches [11, (IQR 2.8–35) mm vs. 11 (IQR 2.3–36) mm, *p* = 0.745].

### Secondary outcomes

The robotic and laparoscopic groups had similar rates of 30-day mortality (0.7% vs. 0.9%, *p* = 0.405), 90-day mortality (1.5% vs. 1.8%, *p* = 0.333), and unplanned 30-day readmission (6.6% vs. 6.2%, *p* = 0.477). Robotic surgery was associated with a shorter hospital stay (median: 4 vs. 5 days, *p* < 0.001) and a lower rate of conversion to open surgery (5.8% vs. 13.6%, *p* < 0.001) (Table 3). The odds of conversion to open surgery were lower with robotic-assisted surgery (OR: 0.39, 95%CI 0.34–0.45, *p* < 0.001). The same finding was noted with robotic-assisted LAR (OR: 0.42; 95%CI 0.35–0.49, *p* < 0.001), robotic-assisted APR (OR: 0.35 95%CI 0.26–0.46, *p* < 0.001), and robotic-assisted pelvic exenteration (OR: 0.38, 95%CI 0.17–0.84, *p* = 0.016).

**Table 3:** Outcomes of the matched cohort

Factor	Group	Laparoscopic	Robotic	*p*-Value
Number		5631	5631	
Conversion (%)	No	4867 (86.4)	5307 (94.2)	** < 0.001**
	Yes	764 (13.6)	324 (5.8)	
30-day mortality (%)	No	5171 (99.1)	5181 (99.3)	0.405
	Yes	47 (0.9)	35 (0.7)	
90-day mortality (%)	No	5105 (98.2)	5123 (98.5)	0.333
	Yes	92 (1.8)	73 (1.5)	
30-day readmission (%)	No	5196 (92.9)	5183 (92.3)	0.477
	Planned	40 (0.7)	53 (0.9)	
	Unplanned	352 (6.2)	373 (6.6)	
	Planned and unplanned	5 (0.1)	6 (0.1)	
Median hospital stay in days [IQR]		5 [4, 7]	4 [3, 7]	** < 0.001**
Circumferential resection margin (%)	Negative	4567 (90.6)	4519 (89.5)	0.072
	Positive	473 (9.4)	528 (10.5)	
Surgical margins (%)	Negative	4984 (89.4)	4961 (88.7)	0.317
	Positive	594 (10.6)	629 (11.3)	
Median number of examined lymph nodes [IQR]		15 [12, 20]	15[12, 20]	0.105

IQR, interquartile range. Bold text in *p* value column indicates statistical significance

**Fig. 3:**
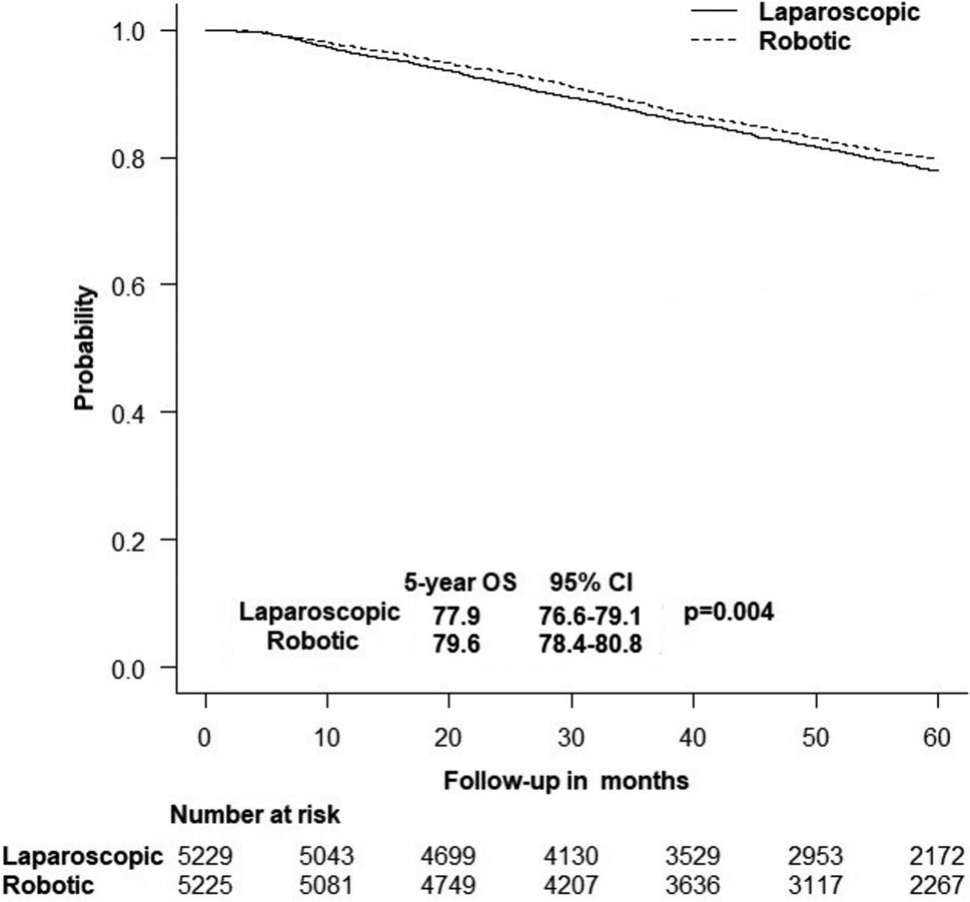
Kaplan–Meier plot showing the difference in overall survival between robotic-assisted and laparoscopic proctectomy

The 5-year OS rate was higher with robotic-assisted proctectomy (79.6% vs. 77.9%, *p* = 0.004) (Fig. 3). When stratified by disease stage and type of proctectomy, the survival benefit of robotic-assisted surgery was noted only in stage III disease (80.3% vs. 78.3%, *p* = 0.014) and low anterior resection (81.8% vs. 79.1%, *p* < 0.001) (Table 4). The robotic-assisted approach was not independently associated with OS of rectal cancer (HR: 0.92, 95%CI 0.83–1.02, *p* = 0.115). Independent predictors of reduced OS were older age, male sex, advanced Charlson score, high-grade adenocarcinomas, mucinous adenocarcinomas, and lymphovascular and perineural invasion. Conversely, private insurance, low anterior resection, and pull-through with coloanal anastomosis were associated with improved OS (Table 5).

**Table 4:** Mean 5-year overall survival of laparoscopic and robotic-assisted proctectomy stratified by stage and type of surgery

Factor	Group	5-year OS (%)	95%CI	*p*-Value
Stage II	Laparoscopic	77	74.8–79.1	0.143
	Robotic	78.4	76.2–80.4	
Stage III	Laparoscopic	78.3	76.7–79.8	**0.014**
	Robotic	80.3	78.7–81.7	
Low anterior resection	Laparoscopic	79.1	77.6–80.6	** < 0.001**
	Robotic	81.8	80.4–83.2	
Abdominoperineal resection	Laparoscopic	72.4	69.2–75.3	0.193
	Robotic	74.4	71.6–77	
Pelvic exenteration	Laparoscopic	65.2	53.1–74.8	0.888
	Robotic	64.2	52.5–73.7	
Pull through coloanal anastomosis	Laparoscopic	83.4	78.9–87	0.445
	Robotic	80.2	75.6–84	

OS, overall survival; CI, confidence interval. Bold text in *p* value column indicates statistical significance

**Table 5:** Multivariable Cox regression analysis of overall survival

Factor	Group	Hazard ratio	Lower 95%CI	Upper 95%CI	*p*-Value
Age		1.04	1.03	1.05	** < 0.001**
Sex	Female	**Ref**	**–**	**–**	**–**
	Male	1.14	1.03	1.27	**0.016**
Race	White	**Ref**	**–**	**–**	**–**
	Black	1.06	0.88	1.27	0.558
	Asian	0.90	0.70	1.17	0.442
	American Indian	0.78	0.32	1.89	0.588
	Other	0.76	0.49	1.18	0.242
Charlson Deyo Score	0	**Ref**	**–**	**–**	**–**
	1	1.28	1.12	1.45	** < 0.001**
	2	1.30	1.05	1.62	**0.017**
	3	1.81	1.42	2.31	** < 0.001**
Clinical TNM stage	II	**Ref**	**–**	**–**	**–**
	III	1.07	0.96	1.19	0.237
Grade	Low	**Ref**	**–**	**–**	**–**
	High	1.34	1.15	1.56	** < 0.001**
Histology	Adenocarcinoma	**Ref**	**–**	**–**	**–**
	Mucinous adenocarcinoma	1.70	1.38	2.10	** < 0.001**
	Signet ring cell carcinoma	1.83	1.14	2.93	0.123
Insurance	Medicaid	**Ref**	**–**	**–**	**–**
	Medicare	0.85	0.69	1.06	0.149
	Other Government	1.15	0.73	1.82	0.556
	Private	0.69	0.56	0.84	** < 0.001**
	Not insured	1.12	0.79	1.60	0.529
Lymphovascular invasion		1.42	1.25	1.61	** < 0.001**
Neoadjuvant radiation		0.94	0.82	1.08	0.378
Perineural invasion		1.76	1.54	2.00	** < 0.001**
Type of surgery	APR	**Ref**	**–**	**–**	**–**
	LAR	0.83	0.74	0.94	**0.003**
	Pelvic exenteration	1.07	0.79	1.45	0.680
	Pull through with coloanal anastomosis	0.75	0.60	0.93	**0.008**
Approach of surgery	Laparoscopic surgery	**Ref**	**–**	**–**	**–**
	Robotic-assisted surgery	0.92	0.83	1.02	0.115

CI, confidence interval; APR, abdominoperineal resection; LAR, low anterior resection. Bold text in *p* value column indicates statistical significance

## Discussion

The present study emulated a randomized clinical trial comparing laparoscopic and robotic-assisted proctectomy for rectal cancer, using the target trial methodology used in recent coloproctology studies [13, 14]. Our study found both approaches associated with similar pathologic and clinical outcomes. However, the robotic approach was associated with lower conversion rates to open surgery, shorter hospital stays, and increased 5-year OS, mainly in stage III disease.

Consistent with the existing literature [15, 16], the current study cohort consisted mainly of men aged ≥ 60 years, most of whom presented with stage III disease and underwent sphincter-saving surgery after neoadjuvant therapy. This observation suggests that the study population of a target trial is similar to that enrolled in prospective clinical trials. Interestingly, the robotic-assisted approach accounted for approximately 60% of patients. This finding is concordant with the observed time trend of increased use of robotic-assisted surgery for rectal cancer [15]. The present study entailed a contemporary cohort as it spanned a recent period (2015–2021) in which the preference for robotic-assisted surgery dramatically increased.

Because this study was not designed as a prospective randomized trial, several imbalances in patient, disease, and treatment characteristics between the two groups were noted. Importantly, robotic-assisted proctectomy was more often performed for men with advanced disease and elevated pretreatment CEA levels. This trend is expected as robotic-assisted surgery is presumed to have a benefit in challenging rectal cancer cases, such as men with narrow pelvises and locally advanced disease. A contemporary NSQIP analysis [17] also showed that robotic-assisted proctectomy was more likely to be performed in men with increased BMI and advanced T-stage rectal cancers, compared to laparoscopic proctectomy. Moreover, imbalances in surgical expertise among surgeons are unknown.

Patients who had robotic-assisted proctectomy in our study more often received neoadjuvant radiation. This observation may be explained by the more advanced disease or better compliance of patients in the robotic group with neoadjuvant treatments. Since patients who had robotic-assisted proctectomy received neoadjuvant therapy more often, this may explain the longer waiting time before surgery. Furthermore, as a previous study concluded [18], performing major procedures via the robotic platform may prolong wait times pre-surgery intervals presumably due to the limited number of available robotic systems.

After accounting for all observed imbalances between the two groups, using the propensity-score matching method, we were able to obtain two groups, balanced for the main patient and treatment confounders. More than 5500 patients were included in each group, yielding a larger sample size than the published randomized clinical trials [6, 7, 9–11] on the subject. The primary outcome analysis showed similar prevalence of positive CRM and surgical margins and a comparable number of harvested lymph nodes between the two approaches. The margin of difference in the primary outcome was ≤ 1%, and thus the lack of statistical significance is aligned with a lack of clinically meaningful differences.

The similar rates of positive CRM in our analysis were supported by the one randomized trial [7] that showed a similar incidence of involved CRM between the two groups (6.1% vs. 5.5%) and an interim analysis of the COLRAR trial, although a subgroup analysis of patients who had preoperative concurrent chemoradiotherapy showed a lower positive CRM rate in the robotic group [6]. Conversely, the REAL trial [11] showed that robotic surgery had a lower positive CRM rate than laparoscopic surgery (4% vs. 7.2%, *p* = 0.023). Furthermore, a recent meta-analysis of RCTs [19] showed a lower risk of positive CRM with robotic-assisted proctectomy (RR = 0.67, 95%CI 0.49–0.91). The comparable numbers of harvested lymph nodes in the two groups in our analysis were supported by the findings of two randomized trials [6, 7] and a meta-analysis [19]. Another pathologic parameter indicating the quality of surgery is the TME grade, which was not assessed in our study but was found to be comparable among laparoscopic and robotic-assisted proctectomy in two clinical trials [6, 7].

The main benefit of robotic-assisted surgery in our analysis was a 60% lower likelihood of conversion to open surgery. Although this benefit was refuted by the ROLARR trial that showed an unadjusted risk difference of 4.1% [9]; several meta-analyses [19–21] reported 47–68% lower odds of conversion to open surgery with robotic-assisted proctectomy compared to laparoscopic proctectomy. It should be noted that the definition and threshold for conversion from minimally invasive to open surgery may vary among surgeons and hospitals [22]. The lack of standardized criteria for what is considered conversion to open surgery may confound the interpretation of the lower likelihood of conversion with robotic surgery.

Overall, the present target trial substantiated the findings of previous small-scale randomized trials on similar clinical and pathologic outcomes of laparoscopic and robotic-assisted proctectomy. Given the known limitations in patient recruitment and follow-up, the large number of patients in each group may be challenging to recruit in a prospective clinical trial, even with a multicenter setting. However, there remains controversy on the effect of robotic-assisted surgery on CRM positivity and conversion to laparotomy. These controversies call for better standardization of the pathologic assessment of rectal cancer specimens and the definition of conversion to laparotomy.

The target trial methodology has several limitations related to the retrospective nature of the data used, missing data, the possibility of misclassifications, and failure to account for other variables that were not available, such as BMI, hospital volume, and the surgeon’s experience. Target trials attempt to emulate RCTs using large observational datasets, which are not feasible when single institutions’ data are used. Despite the lack of granular data on the availability of laparoscopy or robotics, perioperative management, learning curve, and case volume, a target trial adjusts for the main confounders such as demographics, disease stage and characteristics, and insurance type, while providing a large number of patients in each group, sufficient for well-powered and nuanced analyses of secondary outcomes. Although unobserved confounders may have impacted the study results, this impact is not expected to be major because of the narrow margin of difference in the main outcomes. Furthermore, the large number of patients included in the analysis should have helped reduce the risks of an erroneous conclusion. Although we matched the two groups for the facility type, and all hospitals contributing data to the NCDB are CoC accredited, assuming surgeons have completed an adequate learning curve for each procedure, we cannot verify if both laparoscopy and robotic platforms were available at each hospital. Both groups exhibited several significant baseline differences. However, after matching, the two groups were well balanced and matched for demographics, tumor characteristics, and treatment details. Although the effect of residual unobserved confounders should be considered, the study design emulated the outcomes of a randomized trial with selection bias minimized, albeit not eliminated. Finally, the definition of conversion to open surgery may not have been consistent among the hospitals that provide data to the NCDB.

## Conclusions

This target trial found robotic-assisted and laparoscopic proctectomy for rectal cancer associated with similar clinical and pathologic outcomes. Robotic-assisted surgery was associated with fewer conversions to open surgery and shorter hospital stays than laparoscopic surgery.

## Supplementary Information

Below is the link to the electronic supplementary material.

Supplementary file1 (DOCX 16 KB)

## Data Availability

Upon reasonable request to first author.
