# Supplementary material for: Target trial on the outcomes of laparoscopic compared to robotic-assisted proctectomy in stage II–III rectal cancer
Source: Updates Surg. 2025 Oct 16;78(1):117–26. doi: 10.1007/s13304-025-02446-0 (PMC12909341; doi:10.1007/s13304-025-02446-0)
Supplement: Supplementary file 1 — Supplementary file1 (DOCX 16 KB) [file 13304_2025_2446_MOESM1_ESM.docx]

**Supplementary Table.** Characteristics of the cohort studied

| **Factor** | **Group** | **Overall** |
| --- | --- | --- |
| **Mean age in years (SD)** | | 60 (12.42) |
| **Sex (%)** | **Female** | 10194 (38.0) |
|  | **Male** | 16628 (62.0) |
| **Race (%)** | **White** | 22576 (84.8) |
|  | **Black** | 2076 (7.8) |
|  | **Asian** | 1361 (5.1) |
|  | **American Indian** | 150 (0.6) |
|  | **Other** | 460 (1.7) |
| **Ethnicity (%)** | **Hispanic** | 2158 (8.2) |
|  | **Non-Hispanic** | 24244 (91.8) |
| **Charlson Deyo Score (%)** | **0** | 20777 (77.5) |
|  | **1** | 4137 (15.4) |
|  | **2** | 1118 (4.2) |
|  | **3** | 790 (2.9) |
| **Insurance type (%)** | **Medicaid** | 2438 (9.2) |
|  | **Medicare** | 9279 (34.9) |
|  | **Other Government** | 350 (1.3) |
|  | **Private** | 13806 (51.9) |
|  | **Not insured** | 710 (2.7) |
| **Facility type (%)** | **Academic/Research Program** | 9920 (38.9) |
|  | **Community Cancer Program** | 778 (3.1) |
|  | **Comprehensive Community Cancer Program** | 9123 (35.8) |
|  | **Integrated Network Cancer Program** | 5663 (22.2) |
| **Clinical TNM stage (%)** | **II** | 9236 (34.4) |
|  | **III** | 17586 (65.6) |
| **Histology (%)** | **Adenocarcinoma** | 25886 (96.5) |
|  | **Mucinous adenocarcinoma** | 803 (3.0) |
|  | **Signet ring cell carcinoma** | 133 (0.5) |
| **Neoadjuvant radiation (%)** | **No** | 4066 (15.4) |
|  | **Yes** | 22416 (84.6) |
| **Type of surgery** | **Low anterior resection** | 18870 (70.4) |
|  | **Abdominoperineal resection** | 5471 (20.4) |
|  | **Pelvic exenteration** | 544 (2.0) |
|  | **Pull through with coloanal anastomosis** | 1937 (7.2) |
